# Endolysin significantly improves symptoms with atopic dermatitis: bridging the gap from research to clinical practice

**DOI:** 10.3389/fimmu.2025.1667195

**Published:** 2025-10-22

**Authors:** Ling Kui, Jinqun Huang, Guoyun Wang, Jiamin Zhao, Shuangshuang Wang, Xiaoxing Qin, Leifeng Liu, Jianfei Hu, Lianjia Chen, Xingchun Wang, Qing Li, Yuqian Zhao, Yuanyue Tang, Kexu Xiong, Shuxia Zhan, Honghua Ding, JunLing Wang, Hua Cai, Qing Zhang, Xiaoyan Zi, Qiong Deng, Lian Gao, Xuan Wang, Chaowei Zou, Huilin Yang, Yue Xiao, Bin Yang, Bingfeng Leng, Li Zhang, Gang Hu

**Affiliations:** ^1^ Dermatology Department & Medical Cosmetology Department, Shenzhen Qianhai Shekou Free Trade Zone Hospital, Shenzhen, Guangdong, China; ^2^ Shenzhen Beichen Biotech Co., Ltd., Shenzhen, Guangdong, China; ^3^ Hong Kong Rising Biotechnology Co., Ltd., Hong, Kong, Hong Kong SAR, China; ^4^ Suzhou ReadCrystal Biotechnology Co., Ltd, Suzhou, Jiangsu, China; ^5^ 01 Life Institute, Shenzhen, Guangdong, China; ^6^ Dermatology Hospital, Southern Medical University, Guangzhou, Guangdong, China; ^7^ The People’s Hospital of Longhua, Shenzhen, Guangdong, China; ^8^ Zhongshan School of Medicine, Sun Yat-Sen University, Guangzhou, Guangdong, China; ^9^ Peking University Shenzhen Hospital, Shenzhen, Guangdong, China; ^10^ Department of Dermatology and Venereology, West China Hospital, Sichuan University, Chengdu, Sichuan, China; ^11^ Functional Microbiology R&D Center, Research Institute of Tsinghua University in Shenzhen, Shenzhen, China

**Keywords:** atopic dermatitis, *S. aureus*, inflammatory cytokines, endolysin, Staphyrase^®^, phage

## Abstract

**Background:**

Atopic Dermatitis (AD), a chronic inflammatory skin disease characterized by pruritus, dryness, redness, edema, scratching, and lichenification, ranks as the leading cause of non-fatal skin disease burden globally. Current therapeutic strategies for AD primarily act by inhibiting inflammatory pathways, yet largely fail to address *Staphylococcus aureus* (*S. aureus*) control unless exudative lesions are present. However, concerns over treatment-related adverse effects, long-term safety profiles, and emerging drug resistance underscore the remaining substantial unmet clinical needs in this field.

**Objectives:**

To evaluate the safety and efficacy of endolysin gel in treating AD.

**Methods:**

An infection-driven dermatitis model with AD-like features was established. Following treatment with Staphyrase^®^ or in other control groups, skin disease severity scores, *S. aureus* CFU, and key inflammatory cytokines were assessed. An open-label, single-center, investigator-initiated clinical study (ChiCTR25001192) was conducted in which participants, who received the endolysin gel twice daily, underwent follow-up assessments at baseline, treatment weeks 1 and 2, with an optional extension up to 3 months.

**Results:**

Statistically significant reductions in skin lesion scores, *S. aureus* load, and AD-related immune mediators (i.e., IgE, TSLP, IL-33) were observed in the Staphyrase^®^ group relative to the model group. All 20 enrolled adult subjects completed the clinical study, with no tolerability issues reported, indicating a favorable safety profile of the endolysin gel. Compared to baseline, EASI, SCORAD, IGA, VAS, and DLQI scores demonstrated significant decreases at both Day 7 and Day 14 (all P < 0.05). Notably, Participant No. 11, who underwent extended follow-up until Week 8, exhibited substantial improvements in redness, lichenification, severe scratching, oozing, and dryness. The Endolysin gel showed consistent safety and efficacy in improving both acute and chronic AD lesions.

**Conclusions:**

Topical endolysin gel is a well-tolerated, effective, and promising agent for the treatment and proactive maintenance of mild-to-moderate AD in adults.

## 1 Introduction

AD is a chronic inflammatory skin disease characterized by symptoms such as persistent pruritus, dryness, redness, swelling, scratching, and papulation. It constitutes the leading cause of non-fatal skin disease burden worldwide. Notably, epidemiological studies have documented a steady increase in AD prevalence across developed nations, with childhood rates reaching as high as 10-20% in specific populations (1) AD imposes a heavy social and psychological burden on patients due to its recurrent, intractable pruritus, and lack of curative therapies. The chronic pruritus leads to sleep disturbances, severely diminishing quality of life and contributing to heightened risks of depression, anxiety, and even suicidal ideation (2).

*S. aureus* is a prevalent colonizing bacterium in both AD lesions and normal-looking skin. The toxins and enzymes released by *S. aureus* can destroy the skin barrier. Enterotoxins act as superantigens to directly activate lymphocytes, inducing or exacerbating dermatitis while diminishing tissue responsiveness to hormones, thereby compromising therapeutic efficacy (3). A meta-analysis of 95 observational studies based on culture methods revealed that *S. aureus* was present in 70% of patients with AD in skin lesions, compared to 39% in non-lesional skin of patients or healthy controls (4). In another cohort study, *S. aureus* was isolated from 69.7% of eczematous lesions and 42.4% of non-lesional skin in patients with atopic dermatitis. Furthermore, the colonization rate was 53% in mild cases but reached 100% in those with moderate and severe AD patients (5). A further investigation involving 60 children with atopic dermatitis, a similar trend was observed, with *S. aureus* colonization rates proportional to disease severity: 51.43% in mild cases, 77.78% in moderate cases, and 100% in severe cases (6). In a prospective observational study of culturable bacteria on the skin of children from birth to 2 years observed *S. aureus* colonization preceding clinical AD onset (7). Further research has shown that multiple factors are involved in the colonization of *S. aureus* on AD skin, including enhanced bacterial-keratinocyte adhesion, antimicrobial peptide deficiency, decreased levels of filaggrin and filaggrin degradation products, Th2/Th17 cytokines overexpression, microbiota dysbiosis, and altered lipid profiles (8). The V8 protease expressed by *S. aureus* can directly trigger sensory neurons in the skin by activating protease-activated receptor 1, which is also one of the important causes of skin itching (9). In addition, bacterial virulence factors such as phenol-soluble modulin α (PSMα) induce proinflammatory cytokine expression in human keratinocytes and tape-stripped mouse epidermal models (10). Meanwhile, δ toxins promote mast cell degranulation and IgE elevation, amplifying cutaneous inflammation (11). *S. aureus* also expresses several other molecules that enhance symptom intensity, including phenol-soluble modulins (stimulate keratinocytes to release cytokines), protein A (triggers keratinocyte inflammatory responses), superantigens (induce B cell expansion and cytokine release), and proinflammatory lipoproteins (12). Collectively, these findings underscore *S. aureus* as a key pathogen in AD pathogenesis, driving disease progression through barrier disruption, immune hyperactivation, and therapeutic resistance.

Endolysins, bacteriophage-encoded enzymes, exhibit potent bacteriolytic activity against *S. aureus* (13). These enzymes selectively hydrolyze peptidoglycan in bacterial cell walls via Cell Wall-Binding Domains (CBDs), enabling species-specific targeting without disrupting commensal microbiota-a critical advantage over broad-spectrum antibiotics. For instance, recombinant endolysins such as LysM9 demonstrate robust anti-SA activity, including methicillin-resistant strains (MRSA), even under *in vitro* skin-mimetic conditions (14). In addition, endolysin acts on the cell wall surface without penetrating bacterial cytoplasm, thereby circumventing the activation of resistance-conferring genetic pathways (15).

In conclusion, patients with AD are seeking medications that are safe, non-irritating, long-lasting, cost-effective, and suitable for long-term proactive maintenance therapy. Thus, the development of endolysin-based interventions for AD represents a promising and innovative strategy, addressing unmet needs through targeted antimicrobial activity, minimal microbiota disruption, anti-inflammatory and favorable safety profiles. Accordingly, this study aimed to evaluate the safety and efficacy of a novel endolysin-based topical gel in the treatment of *S. aureus*-colonized AD through an integrated assessment of its *in vitro* antibacterial activity, efficacy in murine models with AD-like features, and an initial clinical investigation.

## 2 Results

### 2.1 *In vitro* results

**Figure 1:**
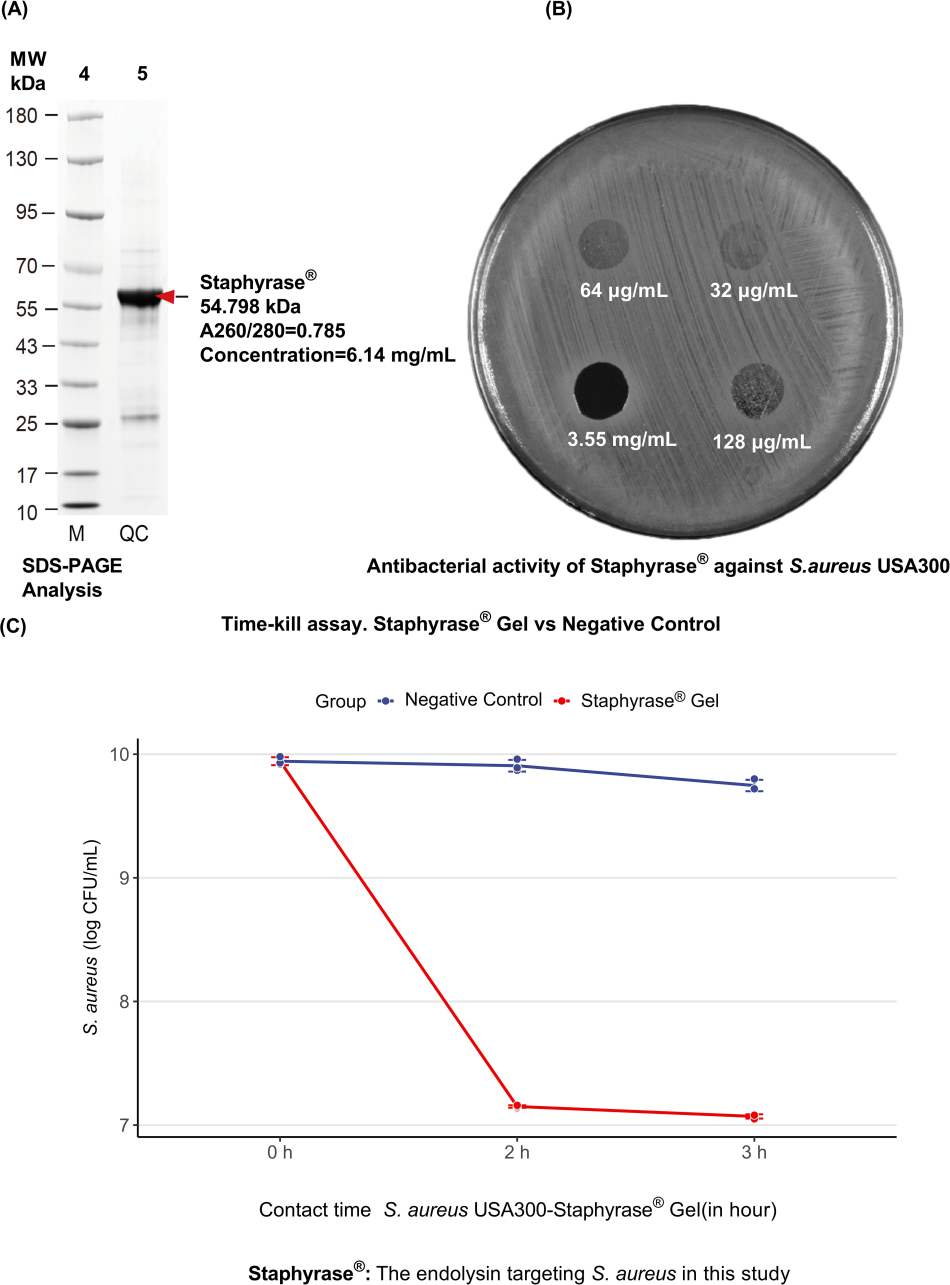
Characterization and antibacterial activity of Staphyrase^®^ against *S. aureus* USA300. **(A)** SDS-PAGE Analysis of Staphyrase^®^ Purity. The quality control (QC) sample shows a single band at approximately 54.798 kDa, corresponding to the expected molecular weight of Staphyrase^®^. The A260/280 ratio was 0.785, and the concentration was measured at 6.14 mg/mL. **(B)** Antibacterial Activity of Staphyrase^®^. The protein was applied at various concentrations: 3.55 mg/mL (concentration), 64 μg/mL, 32 μg/mL, and 128 μg/mL. The increasing zones of inhibition around the protein spots with increasing concentration indicate that the antibacterial effect of Staphyrase^®^ is concentration dependent. **(C)** Efficacy of Staphyrase^®^ Gel against *S. aureus* USA300.

The results illustrate the characterization and antibacterial activity of Staphyrase^®^ against *S. aureus* USA300. SDS - PAGE analysis of the quality control (QC) sample revealed a single band at approximately 54.798 kDa, aligning with the expected molecular weight of Staphyrase^®^. The A260/280 ratio of 0.785 and a measured concentration of 6.14 mg/mL further confirmed the purity and concentration of the sample (**Figure 1A**). **Figure 1B** demonstrates the antibacterial activity of Staphyrase^®^ at various concentrations (3.55 mg/mL concentrate, 64 μg/mL, 32 μg/mL, and 128 μg/mL). The progressive increase in zones of inhibition around the protein spots with rising concentrations clearly indicated that the antibacterial effect of Staphyrase^®^ is concentration - dependent. Furthermore, Staphyrase^®^ was applied to cultures of *S. aureus*, *Staphylococcus epidermidis* (*S. epidermidis*), and *Cutibacterium acnes* (*C. acnes*) isolated from the lesions and the perilesional skin of patients with AD. Lytic activity was observed exclusively against clinical AD-derived isolates of *S. aureus*, with no detectable activity against commensal *S. epidermidis* or *C. acnes* (**Supplementary Figure S1**). These results clearly demonstrate the specificity and safety profile of Staphyrase^®^. And **Figure 1C** evaluates the efficacy of Staphyrase^®^ Gel against *S. aureus* USA300. The negative control maintained a high level of *S. aureus* (log CFU/mL), while the Staphyrase^®^ Gel showed a sharp decline in *S. aureus* concentration at 2 hours of contact time, highlighting its potent antibacterial activity over time.

### 2.2 *In vivo* efficacy of Staphyrase^®^ Gel in a murine AD model

**Figure 2:**
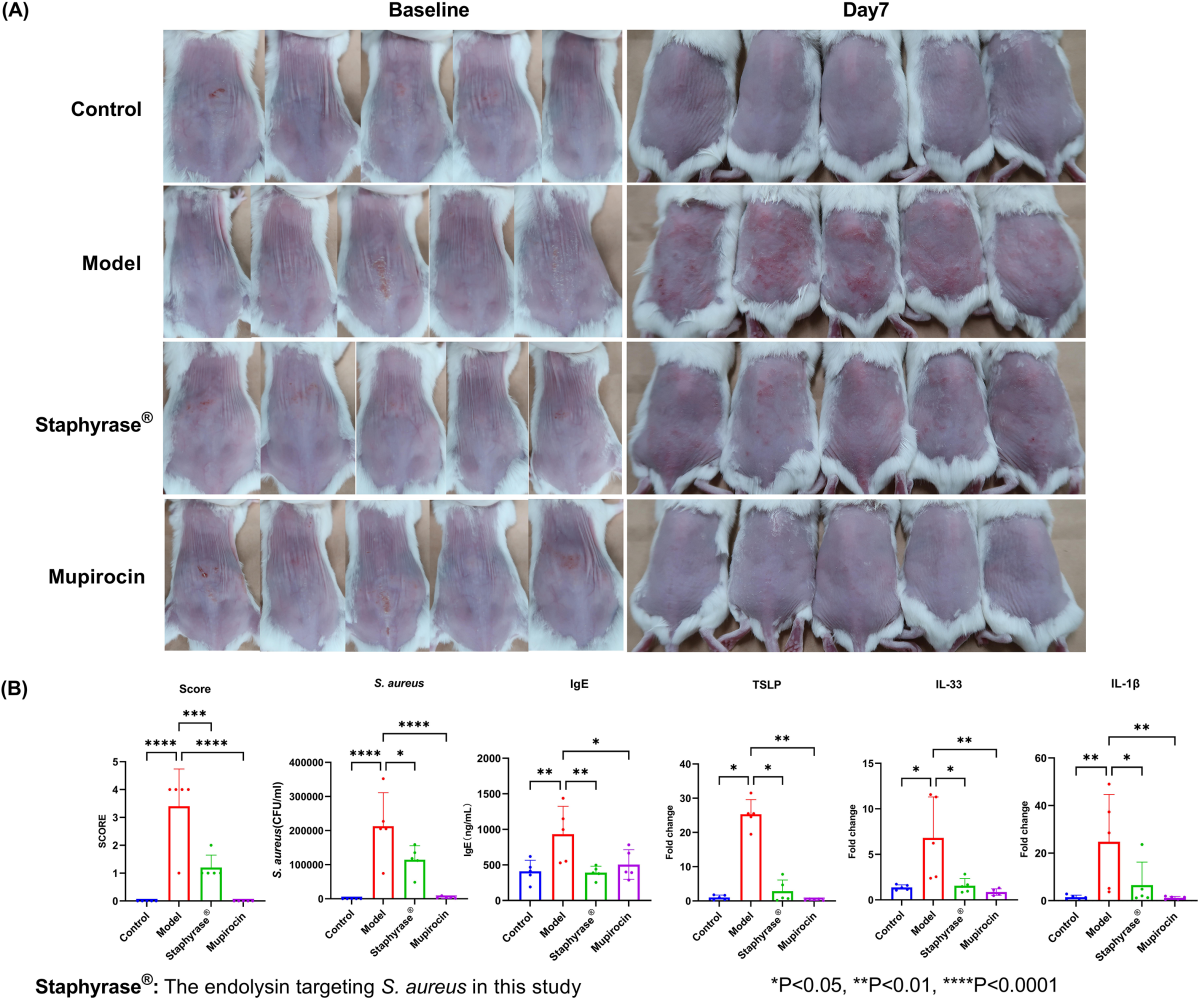
Therapeutic effects of Staphyrase^®^ endolysin BALB/c mice AD model. **(A)** Staphyrase^®^ endolysin treatment demonstrates significant lesion improvement compared to the Model group. **(B)** Staphyrase^®^ endolysin reduces inflammatory factors in BALB/c mice AD model. (Statistical significance is noted with asterisks. *p<0.05, **p<0.01, ***p<0.001, ****p<0.0001).

The therapeutic effects of Staphyrase^®^ endolysin on AD were using a BALB/c mouse model of infection-driven dermatitis with AD-like features (Ethics Number: 01AP202407-02). As depicted in **Figure 2**, representative images of the dorsal skin lesions alongside quantitative analysis of inflammatory markers are presented. For the clinical improvement of skin lesions, at baseline (Day 0), both the Model group (*S. aureus*-inoculated), Mupirocin group (mupirocin-treated) and Treatment group (Staphyrase^®^-treated) exhibited comparable skin injury phenotypes following tape-stripping-induced barrier disruption and bacterial challenge. By day 7, significant conspicuous differences emerged. The Model group (treated solely with *S. aureus*) developed severe redness, scaling, and edema across the entire treated region. In contrast, the Staphyrase^®^ group demonstrated notable amelioration in skin appearance, with diminished redness and scaling relative to the Model group (**Figure 2A**). The Control group (saline-treated) remained free of pathological changes throughout the study. Regarding the disease severity score, quantitative assessment of skin lesions disclosed a significant diminution in disease severity within the Staphyrase^®^ group as opposed to the Model group (p < 0.01). The Model group displayed the highest score, signifying severe AD - like symptoms, whereas the Staphyrase^®^ group showed scores akin to the Control group, underscoring its therapeutic efficacy. Biochemical analyses of skin homogenates and microbiological analyses of lesional swabs showed that IgE levels (a pivotal indicator of allergic inflammation) and the bacterial load of *S. aureus* CFU were both significantly elevated in the Model group compared to the Control group (p < 0.05). Treatment with Staphyrase^®^ led to a substantial decline in both *S. aureus* CFU and IgE levels (p < 0.05), implying effective control of bacterial colonization and alleviation of systemic allergic responses. In terms of the modulation of inflammatory cytokines, RT-qPCR analysis of skin homogenates revealed significant fluctuations in inflammatory cytokine expression. The Model group exhibited upregulated TSLP levels, a marker of epithelial activation in AD, along with increased IL-33 expression, a key marker associated with Th2-type immune responses. Staphyrase^®^ treatment markedly inhibited IL-33-a key mediator of Th2 immune responses in both psoriasis and AD via the activation of ILC2 cells, T cells, and dendritic cells (16)-as well as TSLP expression compared with the Model group (p < 0.05). The pro-inflammatory cytokine IL - 1β was significantly elevated in the Model group but was considerably reduced after Staphyrase^®^ treatment (p < 0.05) (**Figure 2B**). These findings collectively demonstrate that Staphyrase^®^ alleviates AD-like pathology through multi-level modulation of barrier dysfunction, allergic sensitization, and cytokine-mediated inflammation. Although the mupirocin-treated group showed significant improvement in skin lesions, *S. aureus* burden, and levels of pro-inflammatory cytokines (such as IgE, TSLP, and IL-33) compared to the Model group, the increasing prevalence of mupirocin-resistant *S. aureus* in AD patients raises concerns regarding the long-term use of topical antibiotics (17).

### 2.3 Staphyrase^®^ Gel improves symptoms and EASI with AD

Twenty study participants (aged 18 to 56 years) were included in male or female adults with an AD diagnosis meeting Hanifin criterion (> 3 essential features and > 3 secondary features) and had been present for at least 6 months prior to enrollment (EASI ≤ 21 at enrollment). All study participants completed the study without tolerance problems (Clinical registration Number: ChiCTR2500101921). Efficacy outcomes before and after treatment are presented in **Table 1**.

**Table 1:** Demographic and clinical outcomes of AD patients treated with Staphyrase^®^ Gel: EASI, SCORAD, IGA, VAS, and DLQI scores at baseline, day 7, and day 14.

Patient, n	Female	Male	Mean age (min-max) years
20	10	10	18-56
Index	Baseline Mean ± SD	Day 7 Mean ± SD	Day 14 Mean ± SD
EASI	3.81 ± 2.71	3.16 ± 2.43	2.60 ± 2.05
SCORAD	34.34 ± 12.17	26.18 ± 11.63	23.82 ± 12.99
IGA	2.8 ± 0.95	2.2 ± 0.83	1.95 ± 0.83
VAS	6.1 ± 2.20	4.43 ± 2.67	3.25 ± 2.45
DLQI	9.6 ± 4.43	6.05 ± 3.72	5.25 ± 3.77

**Figure 3:**
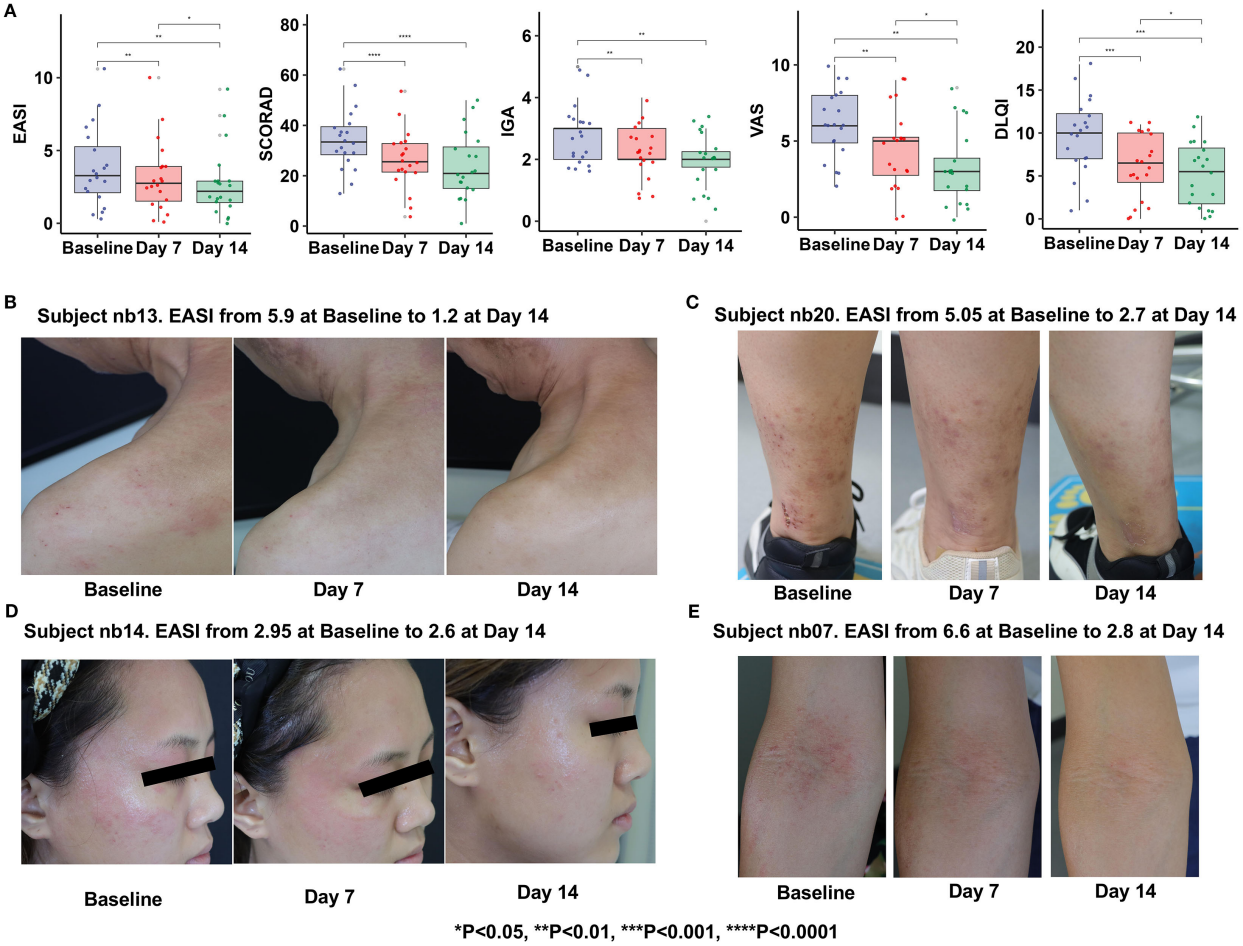
Clinical examinations **(A)** and pictures of lesions treated with the Staphyrase^®^ Gel at Baseline, Day 7, and Day 14 in the Subject nb13 **(B)**, Subject nb20 **(C)**, Subject nb14 **(D)**, Subject nb07 **(E)**. (Statistical significance is noted with asterisks. *p<0.05, **p<0.01, ***p<0.001, ****p<0.0001).

Clinical assessments including the EASI, SCORAD, IGA, VAS and DLQI were measured at Baseline, Day 7 and Day 14 (**Supplementary Table S2**). Nonparametric Wilcoxon signed-rank tests were performed using R software (Version 4.4.1, R Core Team) to evaluate within-subject changes over time. The results showed as follows (**Figures 3A-E**): Compared with the baseline period, all score indexes were significantly decreased at Day 7 (P ≤ 0.05); At Day 14, the scores were further decreased compared with the base-line period (P ≤ 0.05), demonstrating time-dependent efficacy of Staphyrase^®^ Gel (**Figures 3A-E**). It is worth noting that EASI (P = 0.044), VAS (P = 0.017) and DLQI (P = 0.028) scores still showed a significant decreasing trend from Day 7 to Day 14, indicating progressive improvement in lesions severity, pruritus, and patients’ quality of life during the second week (**Table 1**, **Figure 3A**).

**Figure 4:**
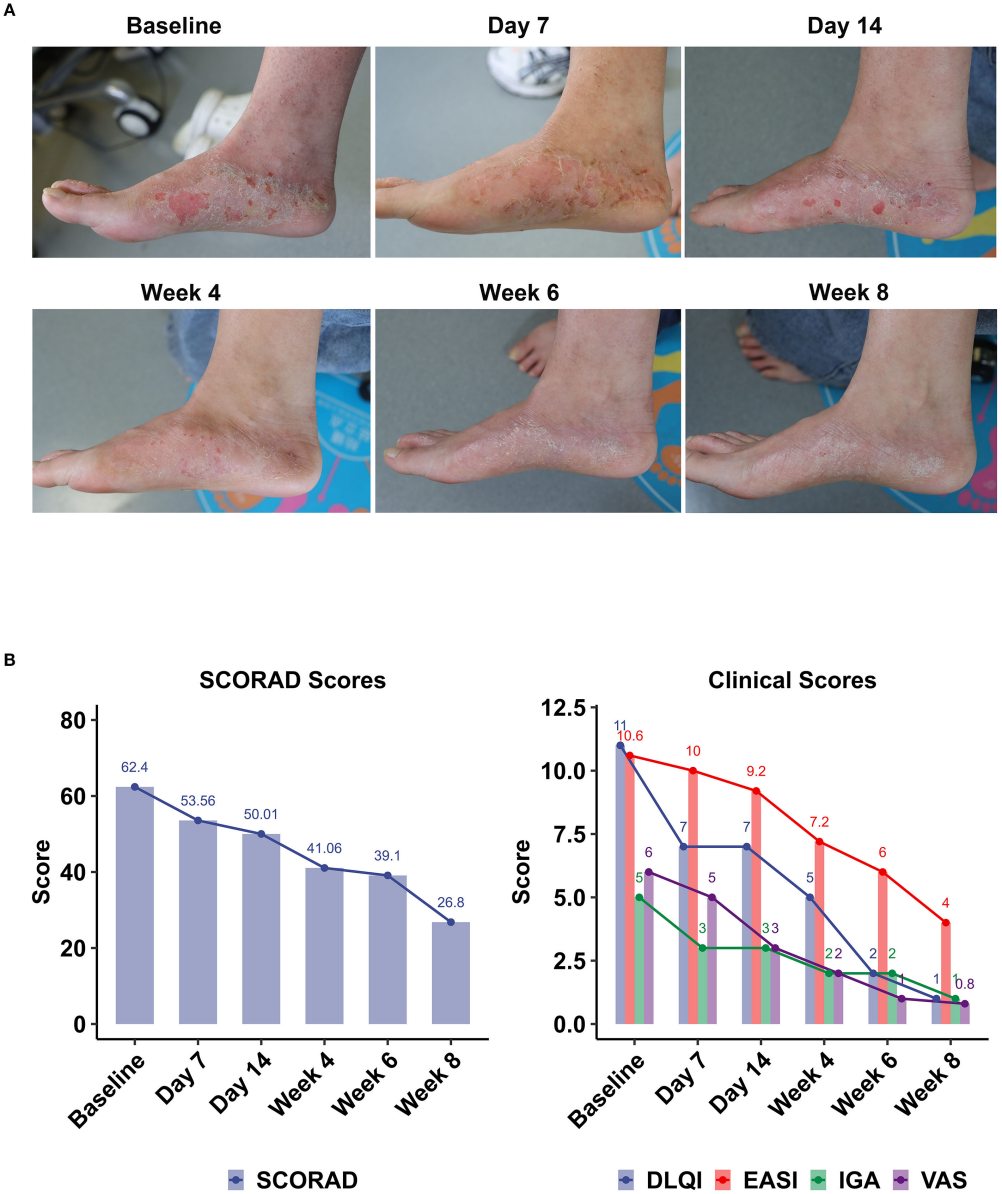
Clinical outcomes following topical Staphyrase^®^ Gel treatment in AD over 8 weeks. **(A)** Pictures of lesions treated with the Staphyrase^®^ Gel at Baseline, Day 7, Day 14, Week 4, Week 6, and Week 8 in Subject nb11. EASI from 10.6 at Baseline to 4 at Week 8. **(B)** Changes in SCORAD, EASI, IGA, VAS, and DLQI scores are shown across consecutive timepoints (Baseline, Day 7 and 14, Week 4, 6, and 8). Notable improvements include SCORAD (62.4 to 26.8), EASI (10.6) to 4, IGA (5 to 1), VAS (6 to 0.8), and DLQI (11 to 1).

Curative effect observation showed that Subject nb13: male. After 14 days of treatment with Staphyrase^®^ Gel, cervical and scapular redness and edema resolved significantly, with EASI decreasing from 5.9 to 1.2 (79.7% reduction) and VAS from 9 to 1. Subject nb20: Female, acute phase. After treatment, Pedal papules, scratching, and redness improved substantially, with EASI declining from 5.05 to 2.7 (46.5% reduction) and pruritus VAS from 10 to 2. The facial papules, edema and redness of Subject nb14, as well as the papules and scratching of the cubital fossa of Subject nb07 were significantly improved, demonstrating the Staphyrase^®^ Gel’s efficacy across acute and chronic AD lesions (**Figures 3**, **4A**).

Case Subject nb11 (female, early onset, family history of atopic disease) showed significant improvement in skin lesions after 8 weeks of treatment with Staphyrase^®^ Gel. The clinical manifestations of pedal redness, scratching and dryness were significantly relieved. Serial assessments revealed sustained improvement across all metrics (**Figure 4B**, **Supplementary Table S2**):

EASI decreased from Baseline 10.6 to 9.2 at Day 14, further declining to 4.0 at Week 8 (62.26% reduction from Baseline); SCORAD decreased from Baseline 62.4 to 53.56 at Day 14 and 26.8 at Week 8 (Δ-57.05%); IGA decreased from Baseline 5 points (severe) to 3 points (moderate) at Day 14 and 1 point (mostly resolved) at Week 8; VAS decreased significantly from Baseline 6 points to 3 points at Day 14 (Δ-50.0%), and only 0.8 points at Week 8 (Δ-86.7%); The DLQI improved from the Baseline 11 points to 7 points at Day 14 (Δ-36.4%) and finally to 1 point (Δ-90.9%).

6 subjects achieved EASI 50+, 6 subjects achieved SCORAD 50+, and 12 subjects improved DLQI by more than 4 points (**Supplementary Figure S2**). The short-term clinical goal is usually 12 weeks, but we spent 2 weeks, a part of the patient index reached the short-term treatment goal (18, 19).

## 3 Discussion

AD is a chronic inflammatory skin disease characterized by pruritus, dryness, redness, and edema, with a significant impact on quality of life (1, 2). *S. aureus* plays a crucial role in exacerbating AD by colonizing both in lesional and non-lesional skin, leading to inflammation and skin barrier dysfunction (4, 7). The toxins and enzymes released by *S. aureus* can destroy the skin barrier and induce immune responses, contributing to disease severity (3, 8).

Endolysin-based therapies, such as Staphyrase^®^ Gel, emerge as a novel therapeutic approach for AD. Endolysins are bacteriophage-encoded enzymes that selectively target *S. aureus* by hydrolyzing peptidoglycan in bacterial cell walls, enabling species-specific bacteriolysis without collateral damage to commensal microbiota (13, 14). This specificity is crucial as it reduces the risk of developing antibiotic resistance, a significant concern with traditional antibiotics (15). Clinical evidence from this study and prior investigations demonstrates the safety and efficacy of Staphyrase^®^ Gel in treating mild-to-moderate AD, with significant improvements in EASI, SCORAD, IGA, VAS, and DLQI scores (18, 19).

Prolonged use of Staphyrase^®^ Gel may afford a therapeutic opportunity to mitigate inflammation and facilitate epidermal barrier repair. By inhibiting *S. aureus*, the gel can potentially decrease the inflammatory burden and allow the skin barrier to recover, thereby alleviating pruritus and improving quality of life (10, 11). However, this study has limitations. The small sample size and open-label design restrict statistical power and limit generalizability to broader AD populations. Additionally, the short follow-up period (14 days primary endpoint, 8 weeks maximum) precludes assessment of long-term safety, sustained efficacy, and recurrence rates. Future studies should aim to enroll more participants and conduct randomized con-trolled trials (RCTs) to further validate these findings and assess long-term outcomes (18, 19).

Notably, the clinical project’s limitations include its open-label design and limited patient enrollment, which may impact the generalizability of the results. Additionally, longer-term data are particularly critical to determine whether continuous *S. aureus* suppression can disrupt the chronic relapse-remission cycle of AD, a key unmet need in current management. Despite these limitations, the study provides promising evidence for the use of endolysins as a targeted therapy for AD, highlighting the potential for improved patient outcomes and reduced reliance on traditional therapy.

## 4 Materials and methods

### 4.1 Endolysin Gel

Staphyrase^®^ Gel was prepared in a formulation containing 32 μg/ml Staphyrase^®^ Endolysin, sodium alginate, calcium chloride, glycerin, purified water accordance with good manufacturing practice (GMP).

### 4.2 Staphyrase^®^ Endolysin expression and purification of protein

Staphyrase^®^ Endolysin was produced by Escherichia coli (*E. coli*) expression system, recombinant expression and purification of endolysin protein.

Protein purification was performed in four sequential steps: Nickel-nitrilotriacetic acid (Ni-NTA) affinity chromatography was first employed, using an imidazole elution gradient of 20 mM, 50 mM, 100 mM, and 400 mM to isolate recombinant endolysin. This was followed by enzymatic cleavage with PreScission Protease overnight, removing the His-tag, and subsequent reverse Ni-NTA chromatography using gradients of 20 mM, 40 mM, and 400 mM imidazole to deplete uncleaved proteins and residual His-tagged intermediates. Anion-exchange chromatography (Q column) was then applied to remove nucleic acid contaminants. The column was equilibrated with 1×PBS (pH 7.4, 10% glycerol), and the flow-through fraction was collected using stepwise NaCl gradients (300 mM and 1000 mM) to exclude charged impurities. Final purification involved dialysis using a Lambolide MD55-7–5 dialysis membrane (7 kDa molecular weight cutoff), against a buffer consisting of 1×PBS (pH 7.4), 300 mM NaCl, and 10% glycerol to achieve buffer exchange and concentrate the protein.

This multi-step protocol, consistent with established methods for recombinant endolysin production in *E. coli* systems (Schme^20^), ensured high purity and yield.

For endotoxin removal, the sample was processed using the Kingsley ToxinEraser™ Endotoxin Removal Kit (Cat. No. L00338) with polymyxin resin, followed by quantitative detection via the limulus amebocyte lysate (LAL) chromogenic assay using the Kingsley ToxinSensorTM Chromogenic LAL Endotoxin Assay Kit (Cat. No. L00350). The final Staphyrase^®^ Endolysin preparation was formulated, aliquoted at a concentration of 2–4 mg/mL, and stored at −80 °C to maintain stability.

### 4.3 *In vitro* methods against *S. aureus* and other commensal bacteria

*Staphylococcus aureus* USA300 glycerol stock was retrieved from a -80 °C freezer and inoculated into Tryptic Soy Broth (TSB) medium for activation culture at 37 °C. Following incubation, the culture was diluted 10-fold with phosphate-buffered saline (PBS) and held as the working bacterial suspension. Lyase samples were thawed from -80 °C storage and diluted to final concentrations of 32, 64, and 128 μg/mL using buffer in a biosafety cabinet under light-protected conditions. Sterile disposable swabs were dipped into the diluted USA300 suspension, with excess liquid expressed against the tube wall to ensure uniform inoculation. The swabs were then streaked evenly across Tryptic Soy Agar (TSA) plates. Ten microliters of each diluted sample (including the undiluted stock) were spotted onto the inoculated TSA plates. After air-drying, plates were incubated at 37 °C, and bacterial lysis zones were observed post-culture (20).

*S. aureus* and *S. epidermidis* isolates from AD lesions and the adjacent skin areas were cultured and streaked evenly onto Tryptic Soy Agar (TSA) plates. In parallel, *C. acnes* was cultured on Brain Heart Infusion (BHI) agar plates under appropriate anaerobic conditions. Following incubation, Ten microliters of Staphyrase^®^ or Buffer was spotted onto the inoculated surfaces of the respective agar plates. The air-dried plates were subsequently incubated inverted at 37 °C. The lytic activity was assessed after the incubation period.

To evaluate the efficacy of Staphyrase^®^ Gel against *S. aureus* USA300, the bacterial strain was resuscitated and cultured to the logarithmic growth phase. The bacterial suspension was then washed twice with sterile phosphate-buffered saline (PBS) and adjusted to a final concentration of 8.8 × 10^9^ CFU/mL for subsequent experiments. Next, Staphyrase^®^ Gel and Negative Control Gel were added to the USA300 suspension at a 1:1 ratio (v/v) respectively, followed by thorough mixing and incubation at 37 °C for 2 h and 3 h, respectively. At each time point, 100 μL aliquots were aseptically collected and subjected to 10-fold serial dilution. Appropriate dilutions were spread evenly onto Tryptic Soy Agar (TSA) plates in trip-licate and incubated overnight at 37 °C. Post-incubation, plates containing 30–300 colonies were selected for viable bacterial count calculation using the formula:


$$Viable\;count\;(\frac{CFU}{mL})=\frac{\left[(Average\;colonies\;per\;plate)\times \;Dilution\;factor\right]}{\text{Inoculum volume }(\text{mL})}$$

This approach aligns with standard microbiological practices for assessing bacterial viability (15). The use of endolysins in such experiments highlights their potential as targeted therapies against *S. aureus*, particularly in the context of antibiotic resistance (14).

### 4.4 Establishment of an infection-driven AD-like mouse model

The animal study protocol was approved by the Institutional Review Board of Shenzhen Zero One Life Science and Technology Co., LTD (Ethics Number: 01AP202407-02, 19.07.2024) for studies involving animals. BALB/c female mice at 6–8 weeks were randomly divided into four groups: 1) negative (Control) group (given normal saline by intragastric administration); 2) Staphyrase^®^ Endolysin lyase (Target) group (30 μg/ml); 3) Model group (*S. aureus*, 10^9^ CFU); 4) Mupirocin group (positive control).

On day 0, mice were anesthetized with 2% isoflurane, and the back hair of mice was removed (2 cm×2 cm), and then wiped with alcohol wipes, and finally removed 20 times with pressure sensitive tape. *S. aureus* (10^9^ CFU) was applied to the skin of the back (2 cm×2 cm) of the exfoliated mice in the endolysin lyase group, Model group and Mupirocin group. For mice in negative control group, equal volume of normal saline was applied to the skin of the peel (21).

From Day 1 to Day 7, the negative control group was given 100 μL of normal saline once a day, the target group received 50 μL of Staphyrase^®^ + 50 μL of *S. aureus* (10^9^), the Mupirocin group received 50 μL of mupirocin + 50 μL of *S. aureus* (10^9^), and the Model group received 50 μL of *S. aureus* (10^9^) + 50 μL of normal saline once a day. After Day 7, Swabs from lesions were collected and cultured for the quantification of *S. aureus* CFU. A sterile swab moistened with PBS was used to repeatedly swab the back lesion area (2 cm × 2 cm, >40 times) and was then immersed in 1 mL of TSB for bacterial colony counting. The sampled TSB tube was serially diluted, and 100 µL of the diluted solution was spread onto mannitol salt agar plates. After spreading, the plates were incubated at 37°C for 24 h before colony counting. Following animal welfare and ethical considerations, the mice were deeply anesthetized via intraperitoneal injection of 2.5% avertin (0.2 mL/10 g). Blood was collected via retro-orbital puncture, followed by ethical euthanasia via cervical dislocation. Skin tissues were then harvested. The specific process diagram can be found in **Supplementary Figure S3**.

### 4.5 RT-PCR for pro-inflammatory cytokines

Following euthanasia, skin tissue (2 cm × 2 cm) was harvested from the central region of the dorsal skin lesion. Total RNA was extracted using the RNA isolater Total RNA Extraction Reagent (Novizan, R401-01) according to the manufacturer’s instructions. Complementary DNA (cDNA) was synthesized from 1 μg of total RNA using the PrimeScript RT reagent Kit with gDNA Eraser (Perfect Real Time) (Takara, RR047A), following the provided reverse transcription protocol.

IgE, IL-1β, TSLP and IL-33 inflammatory factors and *S.aureus* CFU were quantitatively analyzed, and Gapdh was used as substrate. The sequence used is shown in the **Table 2**.

**Table 2:** Primers for real-time PCR analysis.

Primer	Upstream	Downstream
IL-1β	AGCCCATCCTCTGTGAC	GCCACAGGTATTTTGTCGTT
TSLP	AGGCGACAGCATGGTTCTTC	CTGGCTTGCTCTCACAGTCC
IL-33	AGCATCCAAGGAACTTCACTT	CCTGGTCTTGCTCTTGGTC
Gadph	AGGCTGTGGGCAAGGTCA	TGGTCCAGGGTTTCTTACTCC

### 4.6 Assessment of dermatitis in mice

Disease severity of dorsal skin lesions in female BALB/c mice was scored as follows: 0 point- Asymptomatic; 1 point - Mild erythema with minimal focal scaling; 2 points - Moderate diffuse scaling and increased erythema intensity; 3 points - Confluent scaling across ≥ 50% of the dorsal area, moderate erythema, and focal edema; 4 points - Severe generalized scaling, confluent intense erythema, and pronounced edema involving the entire dorsal skin (22).

### 4.7 Participants

From May 2025 to July 2025, 20 patients with AD were recruited in Shenzhen Qianhai Shekou Free Trade Zone Hospital. Inclusion criteria: 1) Age 18–70 years old, gender unlimited; 2) According to Hanifin & Rajka AD diagnostic criteria; 3) EASI score ≤ 21 points at screening and baseline. This study was conducted in accordance with the principles of the Declaration of Helsinki, and the protocol was approved by the Ethics Committee of Shenzhen Shekou Free Trade Zone Hospital of 2025-C-004-K02, (ChiCTR2500101921, https://www.chictr.org.cn/showproj.html?proj=267909), and all subjects signed informed consent. All patients applied Staphyrase^®^ Gel twice daily.

### 4.8 Clinical indicator assessment

The EASI score references Hanifin’s article (23), which notes that EASI score= the area score of the head/neck × the total severity score of the head/neck [redness (E) + Induration (Edema)/Papules (I) + Excoriation (Ex) + Lichenification (L)] × 0.1 + the area score of the upper limbs × the total severity score of the upper limbs (E + I + Ex + L) × 0.2 + the area score of the trunk × the total severity score of the trunk (E + I + Ex + L) × 0.3 + the area score of the lower limbs × the total severity score of the lower limbs (E + I + Ex + L) × 0.4.

SCORAD: It comprehensively assesses the lesion area, objective signs, and subjective symptoms.

- Lesion area: Scored according to the percentage of the body surface area (BSA) affected, ranging from 0 (no involvement) to 100 (entire body affected).
- Objective signs: redness, swelling, oozing/crusting, scratching, lichenification, and skin dryness (in non-affected areas). Each sign is graded 0 to 3 according to severity, yielding a total sign score (B) of 0 to 18.
- Subjective symptoms: The degrees of pruritus and its impact on sleep are respectively scored from 0 to 10 points.

The formula for calculating the total score is as follows: Total SCORAD score = A/5 + 7B/2 + C (24). Here, A represents the area score (ranging from 0 to 100), B represents the sign score (ranging from 0 to 18), and C represents the subjective score (ranging from 0 to 20). The total SCORAD score ranges from 0 to 103 points. A score of ≤ 24 points indicates mild AD, 25–50 points indicates moderate AD, and > 50 points indicates severe AD.

The IGA score was evaluated by investigators during each clinical visit using a 6-point scale (0-5) to assess the global clinical signs of participants. Pruritus severity was measured via a 10-cm VAS, where 0 denoted “no pruritus” and 10 signified “severe pruritus”. Participants self-reported pruritus by marking the scale during each visit. The DLQI assessed health-related quality of life across six domains: symptoms and feelings (itch/pain), daily activities, social and leisure activities, work and study, personal relationships, and treatment-related impact. Each item was rated on a 4-point scale (0 = “no impact” to 3 = “extremely severe impact”), with total scores ranging from 0 to 30. Higher DLQI scores reflect greater impairment in quality of life. adverse events were systematically recorded at all follow-up visits, for safety monitoring, AEs were systematically documented at all follow-up visits, including severity, duration, and causality assessment relative to study interventions. Prior to study initiation, all investigators underwent protocol-specific training. Monitoring personnel conducted regular site visits to ensure compliance with regulatory standards, safeguard participant safety and privacy, and validate data accuracy and completeness (18, 19).

### 4.9 Data analysis

Animal indicators were analyzed using GraphPad Prism 9.0.0 software. Multiple comparisons were performed using one-way ANOVA for statistical analysis and Dunnett for comparative testing. The difference analysis of human body index score was based on EASI, SOARAD, IGA, VAS and DLQI scores. For each score, R software Wilcoxon symbolic rank test was applied at Baseline, Day 7 and Day 14 respectively. P < 0.05 was considered statistically significant.

## 5 Conclusions

The use of Staphyrase^®^ Gel, containing endolysins targeting *S. aureus*, emerges as a promising therapeutic strategy for AD. By selectively addressing bacterial colonization and decreasing the inflammatory burden, this novel intervention effectively manages both acute and chronic skin lesions, alleviates pruritus, and improves key clinical outcomes in AD patients.

## Data Availability

The datasets presented in this study can be found in online repositories. The names of the repository/repositories and accession number(s) can be found in the article/**Supplementary Material**.

## Supplementary material

Supplementary Figure 1 Staphyrase^®^ selectively lyses *S. aureus* but does not affect cutaneous *S. epidermidis* or *C. acnes*.

Supplementary Figure 2 Therapeutic Staphyrase^®^ efficacy in murine model of *S. aureus* skin infection.

Supplementary Figure 3 Therapeutic efficacy of Staphyrase^®^ in murine model of *S. aureus* skin infection. Assessment of Staphyrase^®^ Gel efficacy in 14-day treatment.

Supplementary Table 2 Clinical Scores of Subject_nb11 from Baseline to Week 8.

Supplementary Table 3 Clinical Scores of Atopic Dermatitis Patients from Baseline to Day 14.
