# Supplementary figures and images for: Endolysin significantly improves symptoms with atopic dermatitis: bridging the gap from research to clinical practice
Source: Front Immunol. 2025 Oct 22;16:1667195. doi: 10.3389/fimmu.2025.1667195 (PMC12586070; doi:10.3389/fimmu.2025.1667195)

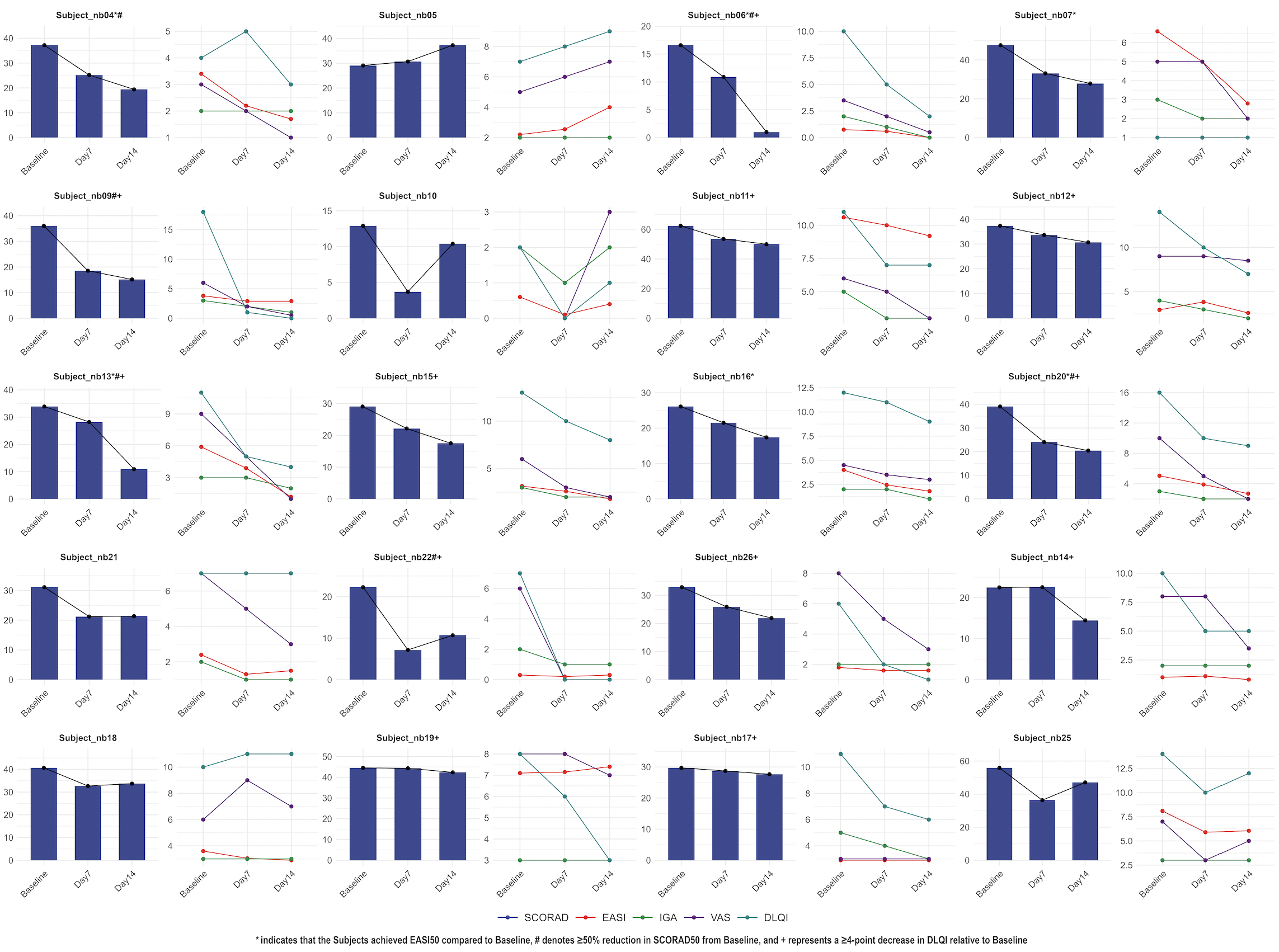

Supplement: Supplementary Figure 2 — Therapeutic Staphyrase® efficacy in murine model of S. aureus skin infection. [file Image2.jpeg]

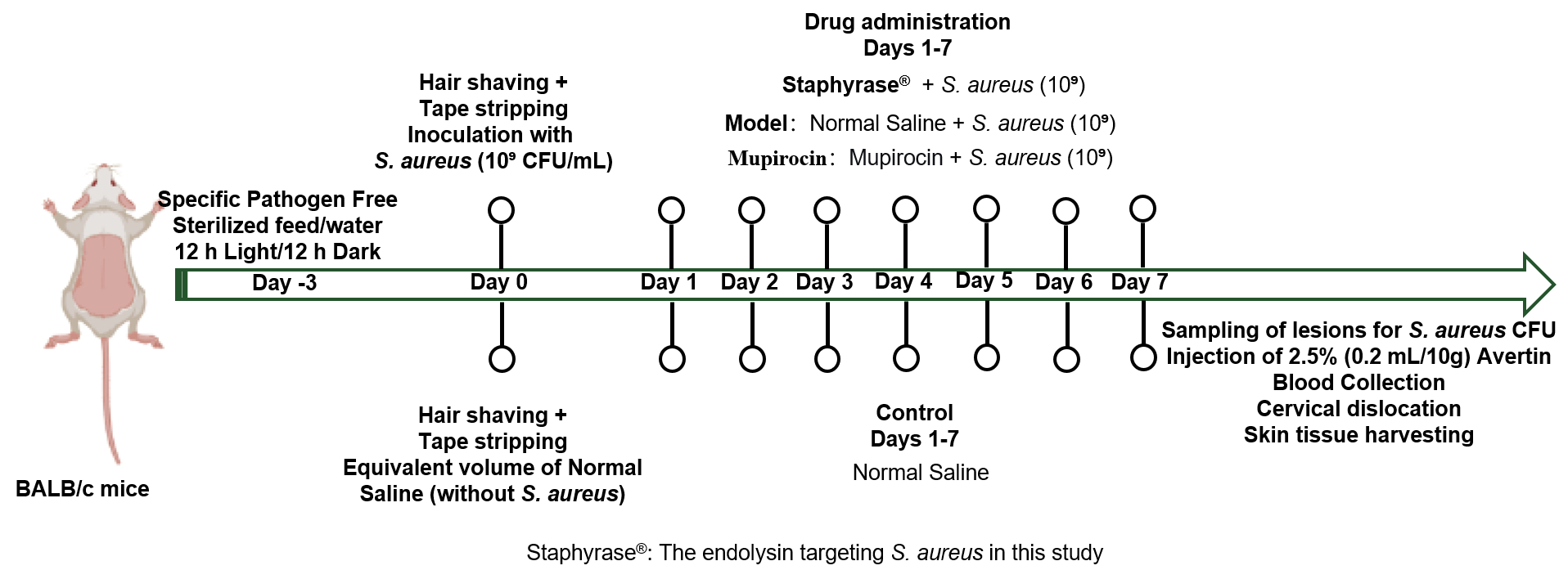

Supplement: Supplementary Figure 3 — Therapeutic efficacy of Staphyrase® in murine model of S. aureus skin infection. Assessment of Staphyrase® Gel efficacy in 14-day treatment. [file Image3.jpeg]

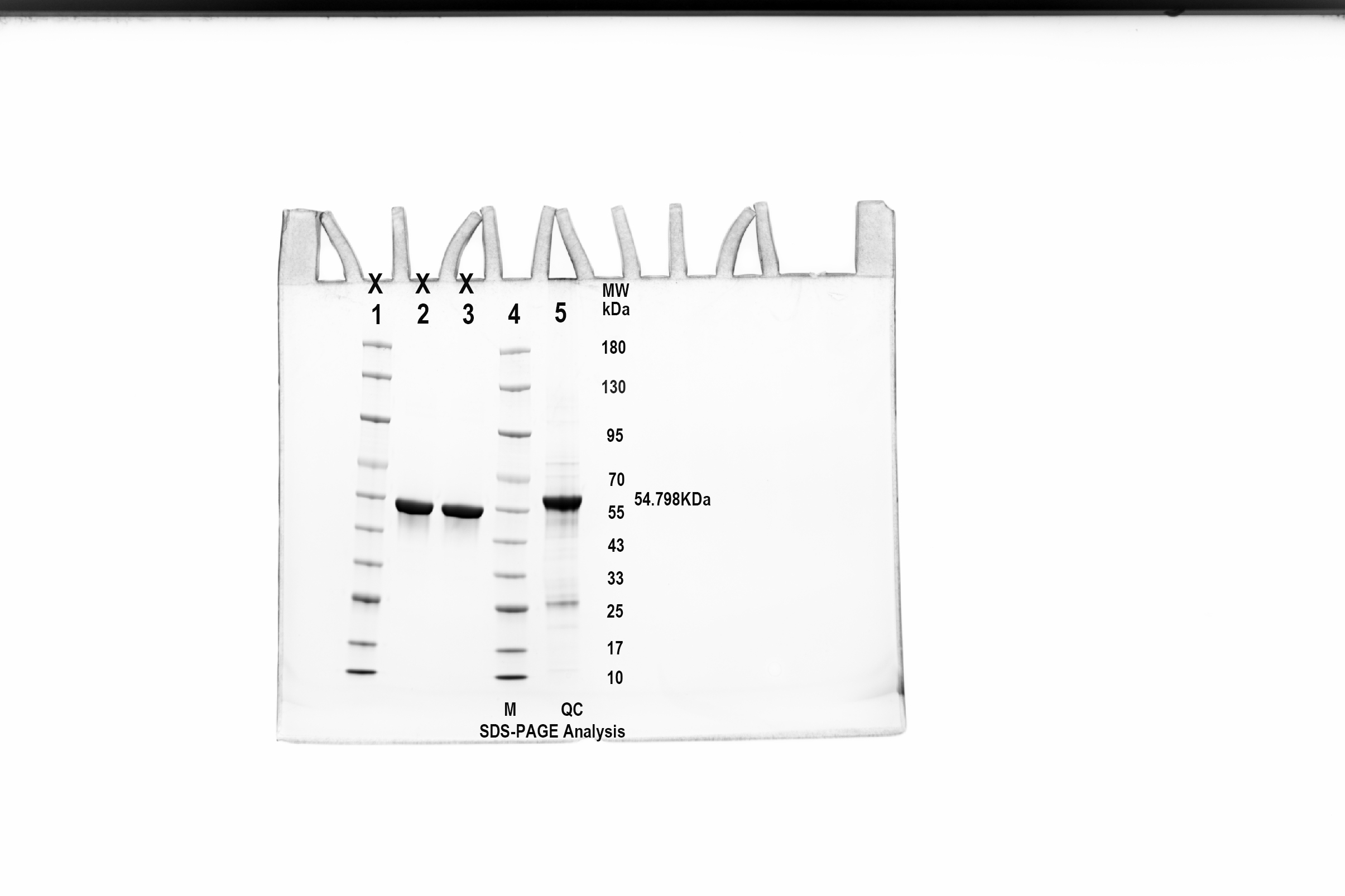

Supplement: Supplementary file 4 [file Image4.jpeg]
