# Supplementary material for: Endolysin significantly improves symptoms with atopic dermatitis: bridging the gap from research to clinical practice
Source: Front Immunol. 2025 Oct 22;16:1667195. doi: 10.3389/fimmu.2025.1667195 (PMC12586070; doi:10.3389/fimmu.2025.1667195)
Supplement: Supplementary Table 2 — Clinical Scores of Subject_nb11 from Baseline to Week 8. [file Table2.docx]

Supplementary Material

# Supplementary Figures and Tables

## Supplementary Figures

**
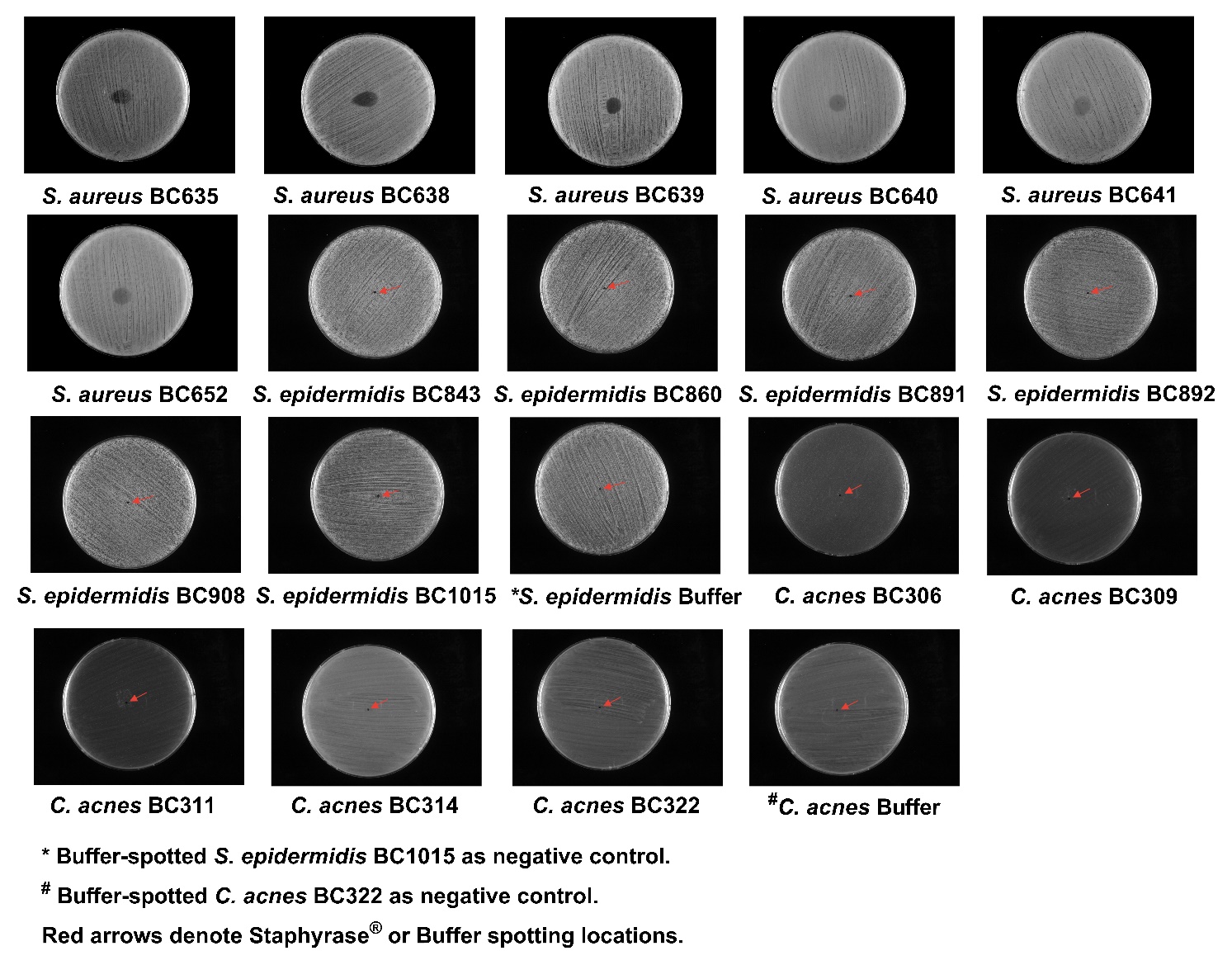
**

**Supplementary Figure 1**: Staphyrase^®^ selectively lyses *S. aureus* but does not affect cutaneous *S. epidermidis* or *C. acnes*. *S. aureus*, *S. epidermidis*, and *C. acnes* were isolated from the lesions and the perilesional skin of patients with AD.


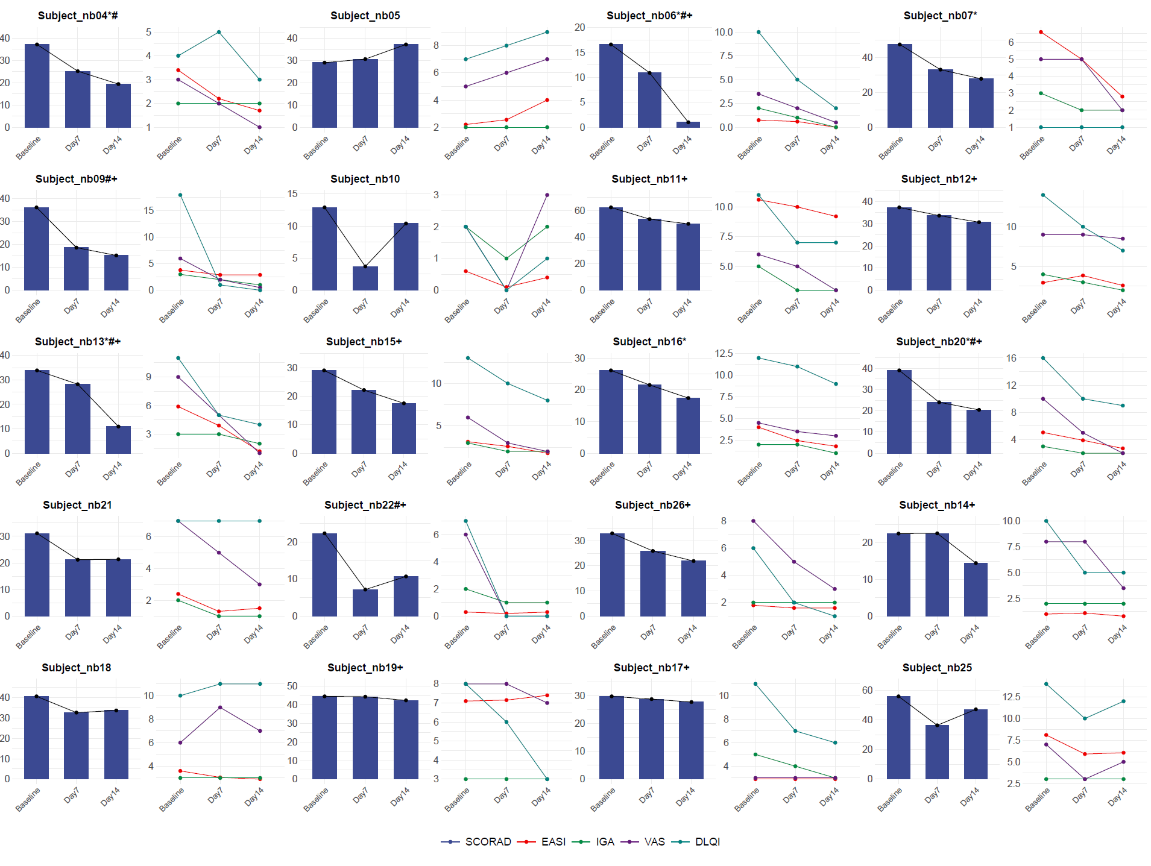


**Supplementary Figure 2**. Assessment of Staphyrase^®^ Gel efficacy in 14-day treatment. Following 14 Days of Staphyrase^®^ Gel treatment, *indicates that the patient achieved EASI 50 compared to Baseline, # denotes ≥ 50% reduction in SCORAD 50 from Baseline, and + represents a ≥ 4-point decrease in DLQI relative to Baseline.


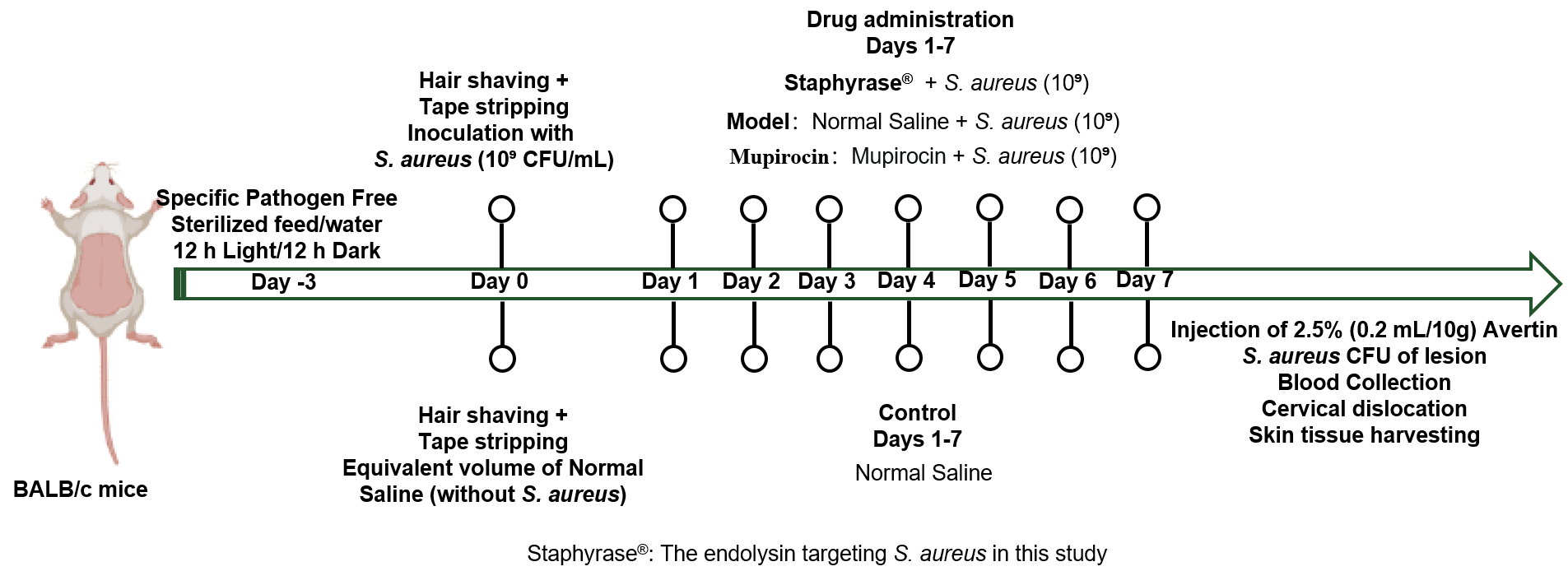


**Supplementary Figure 3**. Therapeutic efficacy of Staphyrase^®^ in murine model of *S. aureus* skin infection. After hair shaving and tape stripping, mice were intradermally inoculated on Day 0 with *S. aureus* (10⁹ CFU/mL; Staphylrase^®^, Model and Mupirocin groups) or sterile normal saline without bacterial inoculation (Control group). From Day 1 to Day 7, daily treatments were administered as follows: the Staphyrase^®^ group received Staphyrase^®^ + *S. aureus* (10⁹ CFU/mL), the Model group received normal saline + *S. aureus* (10⁹ CFU/mL), the Mupirocin group received mupirocin + *S. aureus* (10⁹ CFU/mL), and the Control group received normal saline alone.
